# Higher quality quit-date goal setting enhances quit attempts among quitline callers

**DOI:** 10.18332/tpc/109537

**Published:** 2019-06-06

**Authors:** Benjamin R. Brady, Uma S. Nair, Joe K. Gerald, Nicole P. Yuan, Laurie A. Krupski, Cynthia A. Thomson

**Affiliations:** 1 University of Arizona, Tucson, United States; 2 University at Buffalo, Buffalo, United States

**Keywords:** quit line, goal setting, smoking cessation, tobacco cessation

## Abstract

**INTRODUCTION:**

At tobacco quitlines, coaching and cessation medications are commonly structured around setting a date for making a quit attempt. However, limited literature evaluating this practice suggests that callers do not routinely set quit-date goals. High quality goal setting may increase the frequency of caller quit attempts. In this study, we examine the quality of quit-date goal setting and its association with in-program quit attempts and the timing of callers’ first quit attempt.

**METHODS:**

Using call recordings, we scored the quality of quit-date goal setting among 90 callers enrolled at Arizona Smokers’ Helpline between August and December 2017. The primary exposure was quality of quit-date goal setting assessed using the Lorencatto et al. rating scale. Coding reliability was assessed using Cohen’s kappa. Multivariable logistic regression was used to examine the association between quality of goal setting and in-program quit attempts (>24 h tobacco free).

**RESULTS:**

The mean quality goal setting score was 3.1 (range: -3 to 7). Sixty-nine callers (77%) set a quit date and 39 (43%) made a quit attempt. Compared to callers who experienced low-quality goal setting, the adjusted odds of in-program quitting for high quality goal setting was AOR=3.98 (95% CI: 1.55–10.20) and for making a quit attempt within two weeks OR=6.23 (95% CI: 1.52–25.49).

**CONCLUSIONS:**

Quit-date goal setting is an important element of quitline services and callers benefit from high quality quit-date goal setting. Quitlines should establish quality improvement measures to ensure that coaches are trained to provide high quality quit-date goal setting opportunities to all callers.

## INTRODUCTION

Tobacco use remains the leading preventable cause of death and disease in the US and globally^1^. Quitting is difficult, and most tobacco users make multiple attempts before they successfully become abstinent^2^. Clinical practice guidelines recommend that cessation services, like those offered by quitlines, provide nicotine replacement medication and coaching to educate and support tobacco users to develop coping skills and make positive behavior changes^3^. Quitlines improve individuals’ odds of quitting and have become the standard of care for tobacco cessation^4^.

At quitlines, it is recommended that services are structured around setting a quit date as a goal for callers to become tobacco abstinent^5,6^. Quit dates provide a point of focus for providing behavior change coaching and establishing a schedule to begin using cessation medication. In their first session, coaches should encourage callers to select a day, usually within two weeks, after which they will no longer smoke or use tobacco^7^.

Despite quitlines’ emphasis on setting quit dates, many callers do not. In a research trial, only 32% of quitline participants who indicated an intention to quit in the next 30 days set a quit-date goal^8^. Several factors may influence the decision to set a quit date. However, without high quality services to support quit-date goal setting, callers are not likely to do so. To examine the quality of quit-date goal setting, Lorencatto et al.^6^ developed a rating scale and scored the quality of setting quit-date goals. They found, on average, that the results were ‘low quality’ and only 21% of callers made an in-program quit attempt. However, compared to low-quality goal setting, when high quality goal setting was delivered, callers were more likely to make an in-program quit attempt (OR=2.60, 95% CI: 1.54–4.40). Addressing the quality of goal setting is an important step to improve quitlines’ ability to work with callers and clarify potential discrepancies between their desired behavior change and current behavior.

In this study, we examined one primary and two exploratory analyses. In our primary analysis, we sought to advance the Lorencatto et al.^6^ work by investigating whether the quality of quit-date goal setting was associated with callers making an in-program quit attempt at the Arizona Smokers’ Helpline (ASHLine). We hypothesized that high quality goal setting would be associated with increased odds of making a quit attempt. Recognizing that smokers with mental health conditions (MHC) have historically smoked at higher rates and experienced greater difficulty in quitting^9^, we also examined whether having an MHC modifies the influence of high quality goal setting.

In two exploratory analyses, we further examined whether high quality goal setting is associated with: a) making a quit attempt within two weeks, and b) long-term tobacco cessation. Given that the Lorencatto et al.^6^ quality rating scale prioritizes setting quit dates within two weeks of the first coaching session, in our first exploratory analysis we hypothesized that callers who experience high quality goal setting will make a quit attempt sooner than those who receive low-quality goal setting. In the second analysis, to assess if high quality goal setting is associated with long-term quit outcomes, we examined callers’ odds of being abstinent at the 7 months follow-up. We hypothesized that high quality quit-date goal setting would be associated with increased odds of being abstinent at follow-up.

## METHODS

### Study setting

ASHLine provides behavioral coaching and four weeks of nicotine replacement therapy (NRT) in the form of patches, gum, and lozenges. Callers are assigned a dedicated coach and receive motivational interviewing and elements of cognitive behavioral therapy delivered over seven sessions. Coaches contact enrolled callers weekly to help them identify triggers, set quit dates, develop strategies to manage their urges to smoke, and provide support. In accordance with ASHLine’s client-centered service approach, motivational interviewing techniques are used to explore if and when callers are interested in setting a quit-date goal. Once abstinent, check-in calls are made less frequently. Participation ends when callers: 1) complete the program, or 2) indicate they no longer desire coaching calls or are unreachable (early exit). Callers complete the program at 90 days or once they have received seven coaching sessions. Program participation is not contingent upon setting a quit-date goal.

### Study sample

**Figure 1:**
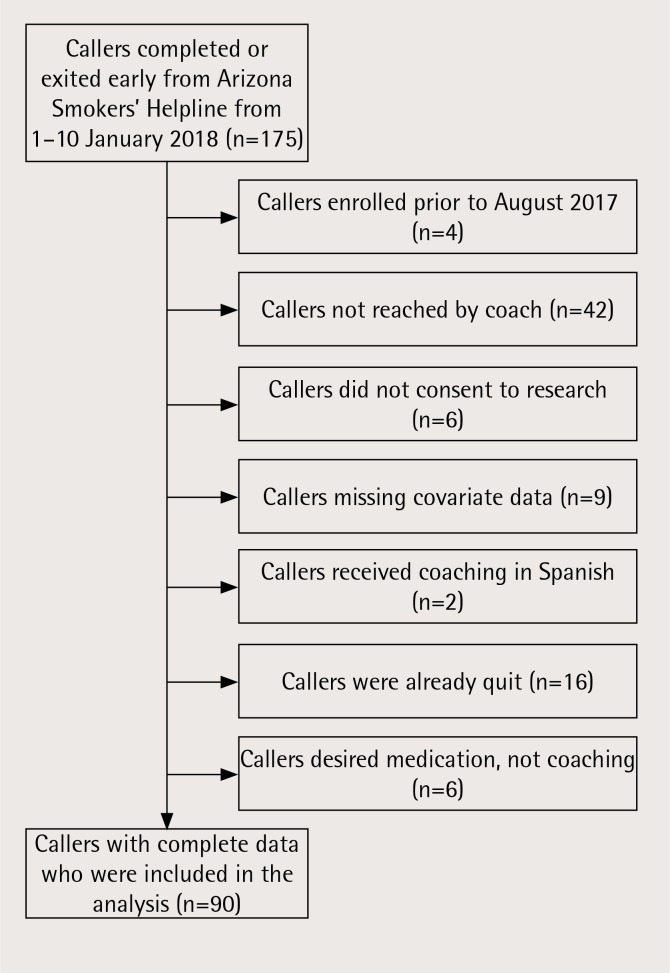
Sample selection and exclusion criteria diagram

We selected callers who completed or exited the program between 1–10 January 2018. Of the 175 callers who completed or exited during this time, 90 met the study’s inclusion criteria. Reasons for exclusion are provided in Figure 1. Because our primary dependent variable involved quitting while in-program, this allowed us to ensure that selected callers were no longer active in the program. The sample was also restricted to callers who enrolled at ASHLine between August and December 2017. In August, ASHLine implemented a coaching protocol that included guidelines for delivering quit-date goal setting congruent with the Lorencatto et al.^6^ quality goal setting scale.

We conducted a power analysis and identified that we needed a sample of at least 88 callers. This estimate assumed one-tail directionality, alpha error probability of 0.05, beta error probability of 0.20, and an expected odds ratio (OR) of 2.0. The OR estimate was based on results from the Lorencatto et al.^6^ study.

**Table 1:** Lorencatto et al.^6^ quality of goal setting rating scale

*Score*	*Quit-date goal setting components*
0	Absence of goal setting (no invitation to set a quit-date goal or behavioral support is delivered by the coach)
1	The coach encourages callers to set a quit-date goal
1	*Agreed quit date is a specific date (dd/mm/yy) OR a specific event (e.g. arrival of NRT)
1	Quit date is scheduled within 14 days following the first coaching session
1	The quit date is scheduled to allow time to first obtain NRT or cessation medication
1	Coach discourages callers from beginning to reduce or cut down smoking/provides advice that reducing is less effective than abruptly quitting on a planned date
1	Coach explains that a quit date entails becoming completely abstinent (e.g. NRT replaces cigarettes, no smoking, not even a puff, after the quit date)
1	Coach provides information and examples about effective behavior changes or NRT/cessation medication use strategies to support quitting and remaining abstinent
-1	Coach encourages callers to reduce or cut down smoking before a scheduled quit date
-1	Set a ‘flexible’ quit date or a goal that is not clearly defined as a specific day/date
-1	The quit date is not scheduled within 14 days following the first coaching session and/or does not allow caller time to obtain NRT or cessation medication

* Twelve codes were used to score the quality of quit-date goal setting. Originally, setting a specific date or a specific event were coded separately. However, they were combined for analysis and callers were awarded a point if they set a specific quit date OR a specific event. This preserved the Lorencatto et al.^6^ original score range (-3 to 7). NRT: nicotine replacement therapy.

Data collected in the enrollment survey and during counseling sessions were de-identified for analysis. We followed STROBE (Strengthening The Reporting of Observational Studies) checklist guidelines^10^ and the study was reviewed and approved by the university’s Institutional Review Board.

### Measures

#### Primary independent variable

Quality of quit-date goal setting is the primary independent variable. We analyzed audio recordings of callers’ first coaching session and measured quit-date goal setting quality using the Lorencatto et al.^6^ rating scale. The scale comprises 10 components that reflect positive or negative elements of quit-date goal setting (Table 1). One point was awarded or subtracted based on the presence of each element. Points were then totaled into an overall score for each caller with a potential range of -3 to 7. To accommodate ASHLine’s process for providing NRT to callers, the criterion for setting a specific quit date was defined as either selecting a date (i.e. dd/mm/yy) or an event (i.e. arrival of NRT). Because NRT sent from ASHLine can take between five and ten days to arrive at callers’ place of residence, this coding assured that in the analysis callers were positively awarded for setting a specific timeline for initiating their quit attempt. Upon conclusion of scoring, we found that the quality goal setting was bimodally distributed with a median quality score of 3.0. For analysis, we dichotomized scores and organized callers into low (-3 to 3) and high-quality groups (4 to 7).

In scoring quality goal setting, one reviewer coded all 90 sessions and a second scored a subset of 15 sessions. This process allowed us to audit the data and assess criteria clarity. External audits are useful for confirming agreement and verifying data trustworthiness^11^. Dual coding only a selected portion of the data also reduces the cost and effort of multiple coding entire datasets^12^. To complete the audit, the first five sessions were coded by two reviewers and compared. Based on observed discordance, criteria were adjusted and the next five sessions were again co-coded and compared. Once the criteria were deemed satisfactory, the primary reviewer scored all remaining sessions. Five additional sessions were randomly selected and cocoded to assess agreement within the full sample. In total, 15 sessions were dual coded. These comprise 180 data points—12 codes for each caller’s session. Eleven codes were based on the Lorencatto et al.^6^ original components plus an additional code: setting a goal around a specific event. To examine intra-rater reliability, 10 sessions (120 data points) were also repeat-coded by the primary reviewer at different time points. We used Cohen’s kappa statistic to compare coding differences^13^. Separate kappa scores are reported for the first five training sessions, the remaining 10 inter-rater audited sessions, and the 10 intra-rater sessions.

#### Dependent variables

The primary dependent variable was making an in-program quit attempt. This was defined *a priori* as being abstinent from tobacco for at least 24 hours, consistent with recommended guidelines^14^. These data were based on caller self-report and were collected by coaches who assessed callers’ tobacco use status in each phone session and recorded the date of their in-program quit attempts. In the primary analysis, quit attempts were classified as a dichotomous outcome (yes/no). In the exploratory analyses, we assessed whether quality of quit-date goal setting was associated with time (in days) until making the first quit attempt. We dichotomized days until abstinent (1–14, ≥15) to reflect the two-week, optimal time frame as defined in the Lorencatto et al.^6^ quality of goal setting rating scale. We also assessed tobacco abstinence at the 7 months follow-up. This was measured as self-reported, 30-day point prevalence abstinence.

#### Covariates

To account for potential differences in callers’ motivation and ability to quit, we selected control variables prior to analysis, based on literature review. In quitline settings, researchers have found that household smoking bans^15^, not having a mental health condition^16^, and higher confidence to quit^17^ are positively associated with quitting. In other settings, living with other smokers^18^, higher nicotine dependence^19^, and lower levels of education^20^, have been found to be negatively associated with cessation outcomes. We also included self-reported age, race, gender and ethnicity, which are known psychosocial determinants of smoking^21^. Because all 90 callers reported an intention to quit smoking in the next 30 days, the variable was not included in the analysis.

We measured comorbidities by callers’ self-report of ever having been diagnosed by a healthcare professional with: asthma, cancer, COPD, diabetes, heart disease, or hypertension (yes/no). Mental health status was similarly assessed as having ever been diagnosed with: anxiety disorder, depression, bipolar disorder, schizophrenia, or alcohol/drug abuse disorder (yes/no). The Fagerström test was used to measure nicotine dependence (low 0–2, moderate 3–5, heavy 6–10)^22^ and home smoking bans were categorized as smoking not allowed anywhere in the home (full ban), smoking allowed in some places (partial ban), and smoking allowed anywhere (no ban). We dichotomized other smokers in the home (yes/no) and callers’ confidence in being able to remain abstinent for a 24-h period. Caller confidence was defined as not confident (not or somewhat confident) and confident (confident, very confident, or extremely confident). Finally, we included a categorized count of callers’ previous ASHLine enrollments (0, ≥1).

### Primary analysis

This study was a retrospective cohort study. We described characteristics for callers who experienced high-quality and low-quality quit-date goal setting (Table 2). We used chi-squared, Fisher exact and unpaired two-sample t-tests to examine differences between them. We used multivariable logistic regression models to examine the adjusted and unadjusted odds and 95% confidence intervals (95% CI) for making an in-program quit attempt by quality of quit-date goal setting. Our *a priori* hypothesis was that, compared to low-quality goal setting, high-quality goal setting would be associated with higher odds of making a quit attempt. To examine potential moderation from having a mental health condition, we used a likelihood ratio test to assess its interaction with quality of quit-date goal setting.

**Table 2:** ASHLine caller characteristics by quality of quit-date goal setting (August–December 2017). Categorical variables displayed as n (%) and continuous variables displayed as mean (SD)

	*All callers n=90*	*High-quality goal setting n=43 (48%)*	*Low-quality goal setting n=47 (52%)*	*p*^*a*^
**Caller demographic, tobacco use history, and program engagement characteristics**				
**Gender**				0.76
Female	55 (61)	27 (63)	28 (60)	
Male	35 (39)	16 (37)	19 (40)	
**Age**				0.46
18–24	4 (4)	3 (7)	1 (2)	
25–44	24 (27)	9 (21)	15 (32)	
45–64	42 (47)	22 (51)	20 (43)	
≥65 years	20 (22)	9 (21)	11 (23)	
Mean (SD)	51.4 (14.8)	52.0 (15.5)	50.8 (14.3)	0.69
**Race**				0.46
White	68 (76)	34 (79)	34 (72)	
Non-White	22 (24)	9 (21)	13 (28)	
**Ethnicity**				0.51
Hispanic	10 (11)	6 (14)	4 (9)	
Non-Hispanic	80 (89)	37 (86)	43 (91)	
**Education**				0.41
High School/GED or less	42 (47)	22 (51)	20 (43)	
Some college or more	48 (53)	21 (49)	27 (57)	
**Any mental health condition**^b^				0.22
Yes	50 (56)	21 (49)	29 (62)	
No	40 (44)	22 (51)	18 (38)	
**Any chronic health condition**^c^				0.23
Yes	56 (62)	24 (56)	32 (68)	
No	34 (38)	19 (44)	15 (32)	
**Nicotine dependence (Fagerström)**				0.19
No/very low (0–2)	16 (74)	9 (21)	7 (15)	
Moderate (3–5)	36 (40)	13 (30)	23 (49)	
High (6–10)	38 (42)	21 (49)	17 (36)	
**Other smoker(s) in the home**				0.23
Yes	34 (38)	19 (44)	15 (32)	
No	56 (62)	24 (56)	32 (68)	
**Home smoking rules**				0.88
Smoking allowed anywhere (no ban)	14 (16)	7 (16)	7 (15)	
Smoking allowed in some places (partial ban)	9 (10)	5 (12)	4 (9)	
Smoking not allowed (full ban)	67 (74)	31 (72)	36 (77)	
**Abstinence confidence for 24 hours**				0.12
Not or somewhat confident	50 (56)	21 (49)	29 (62)	
Confident, very confident, or extremely confident	40 (44)	22 (51)	18 (38)	
**Number of prior ASHLine enrollments**				0.51
0	75 (83)	37 (86)	38 (81)	
≥1	15 (17)	6 (14)	9 (19)	
**Quit-date goal setting and quit attempts Set a quit-date goal in first session**				<0.001
Yes	56 (62)	43 (100)	13 (28)	
No	34 (38)	0 (0)	34 (72)	
**Made an in-program quit attempt**				0.007
Yes	39 (43)	25 (58)	14 (30)	
No	51 (57)	18 (42)	33 (70)	

a Two group t-tests were used to assess continuous variables. Due to low expected values (<5), Fischer exact tests were used to evaluate age, ethnicity, and home smoking rules. Remaining categorical variables were tested using chi-squared.

b Mental health conditions: anxiety, depression, bipolar disorder, alcohol/drug abuse, or schizophrenia.

c Chronic health conditions: asthma, cancer, COPD, diabetes, heart disease, or hypertension. SD: standard deviation.

In a multivariable logistic regression model, we included the caller demographic, tobacco history and program use variables listed in Table 2. This represented our full model. To avoid over-fitting the model, we used backwards selection to remove variables not significantly associated with making an in-program quit attempt. Covariates were excluded with a p-value >0.05. Quality of quit-date goal setting and age remained in the model. This represented our reduced model. We used a gamma test to examine the pairwise association among the independent variables^23^. We used a likelihood ratio test to examine differences between the full and reduced models.

### Exploratory analyses

In the exploratory analyses, we assessed the relationship between quality of quit-date goal setting and making a quit attempt within two weeks of initiating coaching services. For this, we fit a second logistic regression using a subset of callers from the original sample who made an in-program quit attempt (n=39). To keep the independent variable distinct from the dependent, we re-scored the independent variable, quality of quit-date goal setting, by removing the two elements that penalized and rewarded setting long and short quit-date goals, respectively. Beginning with the same set of covariates used in the primary analysis, we again used backwards selection to fit a reduced model. We forced quality of quit-date goal setting into the model and set a stopping rule to drop all other variables with a p-value >0.05. Only quality of quit-date goal setting remained. We repeated these steps to also examine the odds of smoking abstinence among callers reached at the 7 months follow-up (n=33) by quality of quit-date goal setting. As before, only quality of goal setting remained in the reduced model. The full and reduced models for both of the exploratory analyses were compared using a likelihood ratio test. Statistical tests were performed using Stata 15.1 (StataCorp LLC. 2017 College Station, TX).

## RESULTS

We reviewed 90 ASHLine callers’ first coaching session delivered by 15 coaches. The inter-rater Cohen’s kappa for the five training sessions was 0.59. This is interpreted as ‘weak’ agreement^13^. Following adjustment to the coding criteria, the additional 10 inter-rater sessions had a kappa of 0.94 and the intra-rater Cohen’s kappa was 0.92. These represent ‘almost perfect’ levels of agreement^13^. Forty-three callers experienced high-quality quit-date goal setting and 47 low-quality goal setting. The mean quality score was 3.1. While in-program, 69 callers (77%) set a quit-date goal and 39 (43%) made a quit attempt. Among callers who made a quit attempt, 33 (85%) had recorded a quit-date goal and 25 (64%) experienced high-quality goal setting. Compared to those who experienced low-quality quit goal setting, callers who experienced high-quality goal setting were more likely to have set a quit date in their first coaching session (p<0.001) and to have made an in-program quit attempt (p=0.007). No other demographic, tobacco use history or program engagement characteristics were statistically different between the high-quality and low-quality groups (Table 2).

In the adjusted model, callers who experienced high-quality goal setting were more likely (AOR=3.98, 95% CI: 1.55–10.20) to make an in-program quit attempt than those who experienced low-quality goal setting (Table 3). Compared to callers 45–64 years old, those over 65 years were also more likely to make a quit attempt (AOR=3.83, 95% CI: 1.15–12.68). Pairwise association tests showed no association between covariates in the reduced model. We found that MHC did not moderate the association between quality quit-date goal setting and in-program quitting; the likelihood ratio test showed that the interaction term was not significant in the model (p=0.27).

**Table 3:** Adjusted odds ratios (AOR) of in-program quit attempts (n=90)

	*Multivariable model in-program quit AOR (95% CI)*	*p^a^*
**Quality of quit-date goal setting**		
Low	Ref.	-
High	3.98 (1.55–10.20)	0.004
**Age**		
18–24	0.38 (0.03–4.30)	0.44
25–44	1.21 (0.40–3.66)	0.73
45–64	Ref.	-
≥65 years	3.83 (1.16–12.68)	0.03

a Multivariable logistical regression analysis was used to assess the adjusted odds of making an in-program quit attempt; p-values were based on an alpha of 0.05.

In our first exploratory analysis, using the re-scored, quality of goal setting independent variable, we found that among callers who made an in-program quit attempt, the odds of making a quit attempt within two weeks of the first coaching session was greater (OR=6.23, 95% CI: 1.52–25.49) for those who experienced high-quality quit-date goal setting compared to low-quality goal setting. In the second, we explored long-term quit outcomes among 33 callers reached at follow-up (37% response rate). Of those, 14 indicated that they had not smoked in the past 30 days for an intent-to-treat (ITT) quit rate of 16%. The ITT quit rate was 21% for callers who experienced high-quality quit-date goal setting and 11% for those who experienced low-quality goal setting. However, the difference in odds of being abstinent were not statistically significant (OR=2.00, 95% CI: 0.49–8.24) for callers who experienced high-quality compared to low-quality goal setting. As with the primary analysis, we used the reduced model in both exploratory analyses; the likelihood ratio tested showed no difference between full and reduced models for each.

## DISCUSSION

Consistent with the Lorencatto et al.^6^ study, we found that delivery of high-quality quit-date goal setting was positively associated with callers making an in-program quit attempt. Higher quality goal setting was also associated with increased odds of callers reporting a quit attempt within two weeks of their first coaching session. Callers’ long-term odds of being abstinent were not significantly different for those who experienced high-quality versus low-quality quit-date goal setting. Given that the ITT quit rate for high-quality goal setting was almost double the quit rate for low-quality goal setting (21% vs 11%), this finding is likely due to the small and insufficient sample of callers who completed the follow-up assessment at 7 months.

In the Lorencatto et al.^6^ pilot study, using the quality of goal setting rating scale, an average quit-date quality goal setting score of 1.6 was described with 21% of the sample initiating a quit attempt. In our sample, both the quality of quit-date goal setting and the prevalence of quit attempts were higher, with an average quality of quit-date goal setting score of 3.1 and 43% of sampled callers reporting an in-program quit attempt. This difference may reflect ASHLine coaching protocols and routine training that include components from the quality of goal setting rating scale; prior coach training was not described in the Lorencatto et al.^6^ study.

These findings highlight the importance of the quality of goal setting with regard to setting a quit date. They suggest that when quality quit-date goal setting is consistently delivered, a higher portion of callers will initiate a quit attempt. The quality of goal setting rating scale stresses the importance of setting a proximal quit date and combining it with NRT or other cessation medications. These stood out as important elements of quit-date goal setting. Setting a proximal quit date orients callers’ use of cessation medication and provides a point of focus around which to plan subsequent counseling sessions^24^. Because most quitlines limit callers’ number of counseling sessions, timely initiation of this process is important. Early and open discussions around setting quit-date goals may clarify program objectives, normalize callers’ expectations, and facilitate coach and callers’ ability to articulate potential ambivalence around quitting tobacco.

### Limitations and strengths

Strengths of our study include a statistically powered sample size, review of recorded coaching calls in a sample of coaches trained in quit-date quality goal setting, and use of a previously used instrument for assessing the quality of goal setting. While using the quality of goal setting rating scale may introduce bias in scoring, our data suggest this was well-controlled in our sample with high inter-coder and intra-coder reliability kappa scores. Limitations include the self-reported nature of our primary outcomes including quit attempt and timing of quit attempts. This is an observational study based on a small cohort of callers enrolled in a single quitline. Therefore, the results may not translate to other tobacco cessation services or to the general population of tobacco users. The sample may also be biased as 5% of potential participants were excluded due to missing data, although a comparison of demographic and clinical characteristics did not suggest any significant differences.

In interpreting these findings, it is important to recognize that quality of goal setting rating scale rewards elements that relate to coaching and caller characteristics. It captures an experience to which both contribute. For example, the scale accounts for coaches encouraging callers to set a quit-date goal and whether callers agree and select a date. However, the scale and our analyses did not consider all coaching and caller characteristics that may be associated with quit attempts and cessation. Regarding caller characteristics, we did not assess for specific MHCs or NRT use. Related to coaches, our analyses did not account for differences among coaches, including potential variation in coaching skills. While this is less concerning given the large number of coaches included in the study (n=15), it is plausible that if a coach had a lenient attitude toward quit-date goal setting, leniency towards other key coaching tasks may also be demonstrated, such as commitment to applying motivational interviewing techniques. If present, these differences may have influenced the findings, especially among callers whose quit-date goal setting was scored as low quality. Finally, the study was designed to assess the primary research question. The exploratory analyses may have been underpowered due to small sample sizes.

### Future research

We found evidence that high-quality quit-date goal setting is associated with an increased likelihood of making a quit attempt. We suggest four areas where future research may examine this relationship further. First, future work should determine if standardized quitline training emphasizing high-quality quit-date goal setting practices would improve the quality of quit-date goal setting and enhance the frequency of caller quit attempts. It has been shown that instruments like structured checklists increase protocol compliance and outcomes in care delivery settings^25^. It will be important to develop similar instruments and a fidelity monitoring process to ensure routine delivery of high-quality quit-date goal setting.

Second, to facilitate standardized quit-date goal setting, it may be beneficial to explore ways to improve the quality of goal setting rating scale. Measurement instruments vary in how they aggregate score components^26^. Some dichotomize quality by measuring indicators using an ‘all or nothing’ logic. Others equally weight scale elements and create a composite score by summing present elements^27^. The Lorencatto et al.^6^ scale uses the latter method. An additional approach is to weight elements that are regarded as more important than others^28^. This third option may be better suited for measuring the quality of quit-date goal setting. In our study, we identified a bimodal distribution in the quality of quit-date goal setting scores. This suggests that some elements may ‘stick together’. If there are core elements like setting proximal quit dates, future research could assess whether less important elements are dependent upon them. Thus, it may be beneficial to simply construct a scale with fewer components. For example, of the Lorencatto et al.^6^ 10 components, only a few were independently associated with quit attempts. A reduced scale would likely enhance evaluators’ ability to use it to actively monitor program performance. A randomized experimental design and factor analysis may be appropriate to explore this further.

Third, in the Lorencatto et al.^6^ scale, it is assumed that reducing or cutting down one’s smoking prior to a planned quit attempt is negative. This appears to contradict literature that shows little or no difference in outcomes between smokers who quit abruptly compared to reducing prior to a quit date. Findings that quitting abruptly may be superior to cutting down^29^ attenuate when smokers are consciously attempting to reduce. For example, when instructed to reduce, smokers making a quit attempt demonstrate similar^30^ or superior^31^ abstinence outcomes compared to abrupt quitters. This finding holds among studies examining cutting down while using^32^ or prior to using NRT^33^. We do not find a rationale to discourage callers who desire to begin reducing their smoking prior to a quit date. We checked this assumption by repeating the primary analysis using a modified independent variable. The recalculated quality of quit-date goal setting score excluded the components that rewarded and penalized discouraging and encouraging callers to reduce their smoking prior to making a quit attempt, respectively. While still statistically significant, this change (OR=3.07, 95% CI: 1.22–7.70) attenuated the odds of making an in-program quit attempt compared to the original analysis (OR=3.98, 95% CI: 1.55–10.20). To examine this further, future studies should measure callers’ intention and progress in reducing at the time of enrollment and as a behavior change strategy in their coach-facilitated quit plan.

Finally, future studies should further explore the association between quality of quit-date goal setting and long-term quitting and relapse. Studies with larger and more diverse samples would allow researchers to investigate additional caller characteristics and their readiness to change. This would also enable a more detailed view of the relationship between MHC and quit-date goal setting. Despite evidence that having an MHC decreases quitline callers’ likelihood of quitting tobacco^9,16,34^, we did not find that MHC modified the association between quality of quit-date goal setting and in-program quitting. In a better powered, long-term follow-up analysis, it would be possible to explore if individuals with an MHC, controlling for quality of goal setting, experience greater difficulty in remaining abstinent. Future analysis would also benefit from controlling for type of MHC.

## CONCLUSIONS

Quit-date goal setting is an important element of tobacco cessation coaching, though it has not yet been robustly evaluated in the literature. We found that when callers experienced high-quality quit-date goal setting, their odds of making an in-program quit attempt was four times greater than when quit-date goal setting was lower quality. High-quality goal setting was based on the combination of coaches recommending and clients accepting specific goal-setting conditions, like setting a quit date in conjunction with acquiring NRT and adopting additional behavior changes. This approach also translated to higher odds of making a quit attempt within two weeks of the initial coaching call. Future research should explore interventions to promote high-quality quit-date goal setting, including routine monitoring of quit-date coaching and callers’ goal setting practices.
